# AMPK in BCR-ABL expressing leukemias. Regulatory effects and therapeutic implications

**DOI:** 10.18632/oncotarget.413

**Published:** 2011-12-31

**Authors:** Eliza Vakana, Leonidas C. Platanias

**Affiliations:** ^1^Robert H. Lurie Comprehensive Cancer Center and Division of Hematology/Oncology, Northwestern University Medical School and Jesse Brown VA Medical Center, Chicago, IL 60611

**Keywords:** leukemia, cancer, target, oncotarget, AMPK, mTOR, metformin

## Abstract

The abnormal BCR-ABL oncoprotein is a constitutively active tyrosine kinase driving aberrant proliferation of transformed hematopoietic cells. BCR-ABL regulates activation of many mitogenic and pro-survival pathways, including the PI 3'K/AKT/mTOR pathway that controls various effectors and regulates initiation of mRNA translation in mammalian cells. Although tyrosine kinase inhibitors (TKIs) that target the ABL kinase domain have remarkable clinical activity and have dramatically changed the natural history of Ph+ leukemias, resistance to these agents also develops via a wide range of mechanisms. Efforts to target the PI3'K/AKT/mTOR signaling pathway using kinase inhibitors have been the focus of extensive ongoing investigations by several research groups. Here we review the effects of activation of the AMPK kinase, which regulates downstream targeting and inhibition of mTOR. The potential for future clinical-translational applications of AMPK activators such as AICAR, metformin and resveratrol for the treatment of chronic myelogenous leukemia (CML) and Ph+ acute lymphoblastic leukemia (ALL) are discussed.

## BCR-ABL IN CHRONIC MYELOID LEUKEMIA AND ITS TARGETING BY TKIs

Chronic Myeloid leukemia (CML) is defined by the formation and presence of the Philadelphia (Ph) chromosome, which results from the reciprocal chromosomal translocation t(9;22) (q34;q11) [1, 2]. The protein product of the abnormal *Bcr-Abl* fusion gene is the oncoprotein BCR-ABL, which is expressed in CML and Ph+ ALL [2, 3]. BCR-ABL retains the tyrosine kinase ability of c-ABL but, contrary to c-ABL it is constitutively localized in the cytoplasm, resulting in the engagement and activation of multiple pro-proliferative and anti-apoptotic cascades in transformed cells [2, 3]. Among the cellular cascades activated by BCR-ABL there are mitogen activated protein kinase (MAPK) and phosphatidyl inositol 3' kinase/AKT/mammalian target of rapamycin (PI3'K/AKT/mTOR) pathways [4-7].

The identification of imatinib mesylate (STI-571; Gleevec) as a small molecule ATP-pocket inhibitor of BCR-ABL dramatically re-defined the treatment of CML and had a major impact in the survival of patients with CML and Ph+ ALL [8-13]. As this agent targets directly the ABL kinase domain, its introduction in clinical oncology provided a model for potential selective and specific therapeutic interventions in other malignancies with well-defined targets [11, 12]. Imatinib mesylate, along with second-generation tyrosine kinase inhibitors (TKI) such as nilotinib and dasatinib [14-22], have changed the natural history of CML and have provided important treatment options for this leukemia that in the past was uniformly fatal.

Despite such advances in the field, mutations rendering CML and Ph+ ALL patients non-responsive to TKI's have been identified, including the threonine 315 to isoleucine (T315I) mutation and several others, which differentially prevent binding of different TKIs to the active site of the ABL kinase domain, thereby evading inhibition [23]. Beyond mutations in the kinase domain of BCR-ABL, additional mechanisms of resistance exist [23, 24], further complicating the management of such patients. Although identification of new molecular markers may facilitate response prediction to TKIs and allow optimization of their clinical use [25], there is a need for the development of agents that overcome BCR-ABL TKI resistance. This has led to the ongoing clinical development of new TKIs such as bosutinib that has activity against several imatinib-resistant BCR-ABL mutants with the exception of T315I-BCR-ABL [26] and ponatinib that has activity against T315I-BCR-ABL [28]. Beyond efforts to develop inhibitors that can overcome resistance to first and second generation TKIs, another approach of high potential value is the selective targeting and inhibition of cellular effectors downstream of BCR-ABL. As discussed below, the PI 3'K/mTOR cascade is a prime target for such purpose and has been the focus of extensive investigations.

## HYPERACTIVATION OF THE PI3'K/AKT/mTOR SIGNALING PATHWAY BY BCR-ABL

Among the multiple cellular cascades that are activated by BCR-ABL, the PI3'K/AKT/mTOR pathway [5, 28-30] is of particular interest and has been the subject of extensive efforts by many groups in the CML and Ph+ ALL research fields. One mechanism by which the PI 3'K/mTOR pathway is engaged involves increased production of reactive oxygen species (ROS) by BCR-ABL, leading to inhibition of the serine/threonine phosphatase PP1α, a negative regulator of PI3'K/AKT, ultimately resulting in hyper-activation of the pathway [31, 32]. Blocking the PI3'K/AKT signaling in BCR-ABL cells with the pharmacological inhibitor LY294002 results in increased expression of the cell cycle regulator p27^Kip1^ [33] and decreased expression of VEGF and HIF1α [34]. Furthermore, combining pharmacological inhibition of the PI3' kinase with BCR-ABL kinase inhibitors such imatinib mesylate results in enhanced anti-leukemic effects in Ph+ cells *in vitro* [35].

Downstream of the PI3'K/AKT pathway, the mTOR signaling cascade is also hyperactive in CML [5, 7, 36-38]. mTOR is a central regulatory element in the control of mRNA translation in mammalian cells and functions as the catalytic subunit/kinase for two distinct protein complexes, TORC1 and TORC2 [39-45]. These complexes differ by in the mTOR-binding partners that they include, with TORC1 containing Raptor and TORC2 containing Rictor and mSin1 [39-45]. These complexes regulate distinct cellular processes, with TORC1 being the mediator of signals for initiation of mRNA translation and protein synthesis and TORC2 promoting survival pathways and cytoskeletal reorganization [39-45]. Previous studies have established that TORC1 and TORC2 play critical roles in growth and survival of BCR-ABL transformed cells, including myeloid (CML) and lymphoid (Ph+ ALL) cells [36-38, 46-49], underscoring the importance and relevance of the mTOR pathway in the pathogenesis and pathophysiology of Ph+ malignancies. Notably, the ATP-competitive dual mTORC1/2 inhibitors PP242 and OSI-027 have shown potent growth inhibitory and pro-apoptotic effects in a number of BCR-ABL cell lines and primary patient samples [47, 48] and in a mouse Ph+ ALL mouse model [48], suggesting that these or other similar agents with dual targeting capacities against TORC1 and TORC2 may provide a new alternative approach for the treatment of CML resistant to TKIs.

### Effects of AMPK activation on BCR-ABL-transformed cells

Beyond agents that directly target and inhibit TORCs, indirect suppression of mTOR function by modulation of the AMP-activated Protein Kinase (AMPK) pathway may provide an important alternative therapeutic approach [50]. AMPK regulates mTOR signaling both directly and indirectly. This heterotrimeric protein kinase is activated by means of phosphorylation on the Thr172 site of the α-subunit due to an increased AMP:ATP ratio [51]. Once active, AMPK phosphorylates and activates the TSC2 subunit of the TSC1/2 complex, which in turn suppresses Rheb activity, a small G-protein with regulatory functions on mTOR activation [52-54]. In addition, AMPK has been found to directly phosphorylate the Raptor subunit on Ser792, resulting in inactivation of the TORC1 complex [55].

In one of the initial studies in which the effects of modulation of AMPK in ALL cells were assessed, the AMP-analog AMPK-activator compound 5-aminoimidazole-4-carboxamide-1-beta-4-ribofuranoside (AICAR) was shown to exert antiproliferative effects on childhood ALL cells, including the SupB15 cell line which expresses the p185 BCR-ABL fusion protein [56]. In that study AICAR was found to induce phosphorylation of AMPKα on Thr172, resulting in inhibition of proliferation and cell-cycle arrest at the G1 phase, by increasing the levels of the cell cycle regulators p53, p27 and p21 [56]. The effects of AICAR were enhanced by combination with the mTOR inhibitor rapamycin in all different ALL cell lines tested in that study [56], while the growth inhibitory responses were mediated in part by engagement of the p38 MAP kinase pathway. Another study from the same group [57] established that phosphorylation of AKT on Thr308 and Ser473 increases following treatment of ALL cells with AICAR and demonstrated that AKT phosphorylation on Thr 308 is mediated by AMPK-induced IGF-1R activation and phosphorylation of IRS-1. In follow-up experiments, the authors of that work were able to demonstrate that concomitant inhibition of IGF-1R activity using a tyrosine kinase inhibitor and AMPK activation using AICAR resulted in substantially enhanced antileukemic effects [57].

Resveratrol, a naturally occurring substance found in grapes, has been shown to modulate AMPK in BCR-ABL transformed cells and to exhibit antileukemic effects [57-58]. Treatment of either imatinib mesylate-sensitive or imatinib mesylate-resistant CML cells with resveratrol resulted in cell cycle arrest and apoptotic cell death [57]. Notably, among the resistant cells that were sensitive to resveratrol, there were also cells expressing the T315I BCR-ABL mutant [57]. Subsequent studies demonstrated that resveratrol induces autophagic cell death in CML cells by a dual mechanism involving both engagement of AMPK and JNK-mediated overexpression of p62/SQSTM1 [58].

In a recent study [59], we assessed whether modulation of AMPK by AICAR and the FDA-approved anti-diabetic drug metformin [60-62], exert suppressive effects on BCR-ABL transformed cells. In that study [59] we found that both AICAR and metformin induce AMPK kinase activity, as reflected by phosphorylation of ACC and TSC2. The phosphorylation of various effectors downstream of mTOR was compromised upon treatment in all cell lines tested, including cells harboring the T315I mutation. These studies have indicated that the effects of mTOR inhibition by AICAR and metformin occur irrespectively of TKI-sensitivity [59] and further raised the prospect of using AMPK activators in the treatment of Ph+ leukemias refractory to TKIs, including CML and Ph+ ALL.

## CONCLUSIONS AND FUTURE DIRECTIONS

There have been remarkable advances in the treatment of CML over the last decade. With the introduction of first and second generation TKIs in the treatment of CML, the majority of patients achieve complete hematological, cytogenetic and molecular responses [4, 12]. Also, the introduction of imatinib mesylate, nilotinib and dasatinib in the treatment of patients with Ph+ ALL has dramatically improved their outcome. Nevertheless, large numbers of patients eventually become unresponsive to treatment due to mutations that occur either in the TKI-binding kinase domain of BCR-ABL or due to the emergence of other resistance mechanisms downstream of the kinase [24]. Approaches to target cellular effectors of BCR-ABL may provide a complementary/enhancing approach to the use of TKIs and/or help overcome resistance in cases of resistant CML or Ph+ ALL. Importantly, modulation of the AMPK pathway may allow targeting of mTOR in a distinct, non-overlapping way, from mTOR inhibitors and other suppressors of the PI3'K/AKT/mTOR cascade and AMPK activators could be potentially combined with PI 3'K/mTOR inhibitors in the treatment of refractory myeloid leukemias. Notably, the anti-tumor effects of metformin, a commonly used anti-diabetic drug, have been observed in a number of other hematologic malignancies, including acute myeloid leukemia (AML) [63, 64] and various solid tumors, including breast cancer, lung cancer and others [50, 65-77], suggesting that the clinical development of this drug in clinical oncology may offer advantages for a variety of malignancies. In fact, efforts to combine metformin with mTOR inhibitors for the treatment of solid tumors are already underway [78] and similar studies in Ph+ leukemias and other hematological malignancies are probably warranted from the emerging experimental evidence.

**Figure 1:**
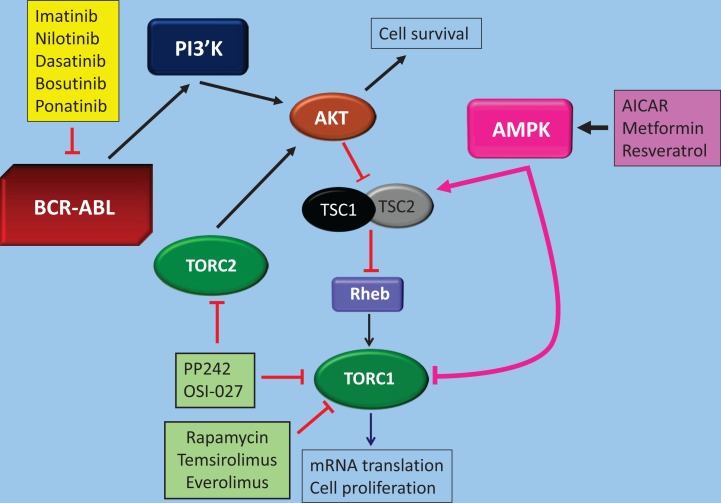
Different target points of AMPK and mTOR pathways in BCR-ABL transformed cells and known pharmacological agents that can be used to target them
